# Mortality and health outcomes associated with non-communicable disease risk factors: Protocol for the IRAN-STEPS Follow-Up Cohort (ISFUC) based on the 2021 national survey

**DOI:** 10.1371/journal.pone.0356802

**Published:** 2026-08-31

**Authors:** Hosein Ataei-Goujani, Sarmad Salehi, Pardis Zarepour, Ozra Tabatabaei-Malazy, Nazila Rezaei, Mina Mirzad, Anis Gharajeh, Samaneh Akbarpour, Yosra Azizpour

**Affiliations:** 1 Non-Communicable Diseases Research Center, Endocrinology and Metabolism Population Sciences Institute, Tehran University of Medical Sciences, Tehran, Iran; 2 Student Research Committee, Shahrekord University of Medical Sciences, Shahrekord, Iran; 3 Department of Epidemiology and Biostatistics, School of Public Health, Tehran University of Medical Sciences, Tehran, Iran; 4 Endocrinology and Metabolism Research Center, Endocrinology and Metabolism Clinical Sciences Institute, Tehran University of Medical Sciences, Tehran, Iran; 5 Sleep Breathing Disorders Research Center (SBDRC), Tehran University of Medical Sciences, Tehran, Iran; Tarbiat Modares University Faculty of Medical Sciences, IRAN, ISLAMIC REPUBLIC OF

## Abstract

Non-communicable diseases (NCDs) account for more than 81% of all deaths in Iran and impose a substantial burden on the national health system. Although Iran has implemented multiple initiatives, including the World Health Organization’s STEPwise Approach to NCD Risk Factor Surveillance (STEPS), to monitor NCD risk factors, critical gaps remain in longitudinal outcome assessment, particularly in the post–COVID-19 period, for which national data are limited. Moreover, in the context of declining fertility rates and demographic challenges in Iran, emerging evidence suggests that metabolic and lifestyle-related NCD risk factors are associated with impaired reproductive health, fertility potential, and childbearing behaviors. Therefore, the assessment of reproductive outcomes within population-based NCD surveillance frameworks addresses an important knowledge gap in Iran. The Iran STEPS Follow-Up Cohort (ISFUC) is established to evaluate mortality, health outcomes related to NCD risk factors, and fertility related outcomes using population-based data from the 2021 STEPS survey. The ISFUC is a population-based cohort study established through follow-up of participants from the nationally representative 2021 Iranian STEPS survey. The 2021 STEPS survey constitutes the baseline assessment, whereas ISFUC represents the subsequent follow-up phase. Baseline assessments were collected through interviewer-administered questionnaires, standardized physical measurements, and laboratory analyses of blood and urine biomarkers. Follow-up began in 4 April 2025, a five-years interval after baseline, and is ongoing. It will be conducted through structured telephone interviews by trained staff after renewed verbal consent. Study outcomes include all-cause mortality, healthcare utilization, injuries, reproductive health indicators, fertility-related behaviors and attitudes, and use of governmental childbearing support policies. Mortality outcomes are validated through linkage with the national death registry. Questionnaire validity was ensured using expert review, the Content Validity Ratio, and the Content Validity Index methods. By linking comprehensive 2021 STEPS baseline data with follow-up information on health, healthcare utilization, mortality, and reproductive outcomes, ISFUC provides a national platform for investigating Iran’s evolving epidemiological and demographic health challenges.

## Background

Non-communicable diseases (NCDs) increased by about 43.9% from 2000 to 2023, rising from approximately 31.1 million deaths to 44.8 million deaths globally. They remained the leading cause of death worldwide in 2023 [1]. NCDs are responsible for over 81% of all deaths in Iran [2,3], imposing the greatest burden on the national health system [4,5]. Notwithstanding the endeavors of policymakers to mitigate NCDs, substantial gaps in risk factor management, including hypertension and high body mass index (BMI), persist. To address these challenges, several national initiatives have been implemented, including the Iran STEPwise Approach to NCD Risk Factor Surveillance (STEPS), a standardized framework developed by the World Health Organization (WHO) for the collection, analysis, and dissemination of NCD risk factor data, implemented in Iran since 2005 [6,7]. In addition, the Iran Package of Essential Noncommunicable Diseases (IraPEN), launched in 2014 as an adaptation of the WHO Package of Essential Non-communicable Diseases (WHO-PEN), aimed to expand access to prevention and care through public education and the integration of hypertension and diabetes services into the primary health care system. Nevertheless, important gaps in implementation remain and require further attention [8]. Moreover, in contrast to earlier control attempts, Iran is currently experiencing an upward trend in multiple NCDs and their associated risk factors. Despite of previous policymaking strategies, projections indicate a substantial increase in major NCDs, including chronic kidney disease (CKD), with the number of affected individuals projected to reach 423,300 by 2030 [9]; and diabetes, projected to affect approximately 8.2 million individuals by 2050 [10]. Moreover, in parallel with the increasing burden of NCDs in Iran, the country is also facing major demographic challenges, including population aging, delayed marriage, declining fertility rates, and reduced willingness of couples to have children, trends that may ultimately result in negative population growth in future decades [11]. Emerging evidence suggests that metabolic and lifestyle-related disorders may adversely affect reproductive health, fertility potential, and pregnancy outcomes [12–17]. Metabolic dysfunction, obesity, diabetes, smoking, and other behavioral risk factors may influence reproductive outcomes through hormonal, vascular, inflammatory, and psychosocial pathways. Their simultaneous longitudinal assessment may therefore provide a more comprehensive understanding of the interrelated NCD and reproductive-health challenges facing Iran.

Cohort studies are essential for generating population-based evidence that enables policymakers to identify NCD-related risk factors and their long-term health consequences, including mortality [18,19]. In Iran, discrete cohort studies have been undertaken in various locations, including northern and central areas, such as the Golestan Cohort Study [20], the Tehran Cardiometabolic Genetic Study (TCGS) [21], and the Yazd Health Study [22]. Nevertheless, prior to the Prospective Epidemiological Research Studies in IrAN (PERSIAN), none of these studies were able to adequately capture the ethnic and public health diversity across the country [5]. Although the PERSIAN cohort study partially addressed these gaps beginning in 2014, its baseline population was limited to 18 specific regions, restricting its representativeness for all subnational populations in Iran [23].

The Iran Cohort Study (ICS) was a large-scale cohort initiative implemented on the basis of the STEPS survey conducted in 2016 [24]. The ICS cohort sought to address limitations of the PERSIAN cohort by enabling analyses at both national and subnational levels using large-scale population-based data [24]. However, ICS was anchored to the 2016 STEPS survey [25]. Compared with the 2016 round, the 2021 STEPS survey, eighth round, introduced substantial enhancements, including the addition of variables such as estimated glomerular filtration rate (eGFR), urine albumin-to-creatinine ratio, and cancer screening indicators, and was conducted in the post–COVID-19 period, a context that markedly affected NCD prevention, care, and management [26,27]. Moreover, the current study solely uses telephone-based interviews instead of ICS, which offer substantial advantages for policymaking, including cost-effectiveness, and easier implementation, particularly in low-income countries [28–31]. In addition, the study incorporates a broader range of outcome domains, encompassing reproductive health and fertility, childbearing patterns, and attitudes toward population growth, alongside conventional NCD outcomes. Specifically, leveraging the STEPS platform for longitudinal follow-up provides a unique methodological opportunity to integrate standardized cardiometabolic risk assessment with reproductive and behavioral outcomes in a nationally representative population to address relative current knowledge gap in Iran. Accordingly, the present study aims to evaluate health outcomes and trends in NCD risk factors through the IRAN-STEPS Follow-Up Cohort (ISFUC), implemented using the population-based 2021 STEPS survey.

## Materials and methods

Informed verbal consent will be obtained via telephone at the start of each encounter prior to initiation of the interview. At the beginning of each call, the interviewer will introduce themselves and clearly explain the study objectives, procedures, expected duration, and intended use of the data. Moreover, participants will be informed about confidentiality, voluntary participation, their right to withdraw at any time, and their option to decline any question. After providing this information, participants will be asked for their verbal consent. Thereafter, verbal consent will be documented electronically within the study data collection system by the interviewer at the time of participant agreement before proceeding with the questionnaire; consequently, only upon receiving their agreement, will the interviewer proceed with the questions. Data will be securely stored and will be used exclusively for scientific purposes. Moreover, all study procedures, including use of verbal informed consent for this telephone-based follow-up study, have been reviewed and approved by Ethical Committee of the National Institute of Health Research (NIHR), Tehran University of Medical Sciences, Tehran, Iran (ID: **IR.TUMS.NIHR.REC.1403.021**) and Research Ethics Committees of Endocrine & Metabolism Research Institute, Tehran University of Medical Sciences, Tehran, Iran (ID: **IR.TUMS.EMRI.REC.1403.169)**.

### Study design and baseline cohort population

The ISFUC is a population-based cohort established through follow-up of participants from the 2021 STEPS survey. The STEPS 2021 survey provides the baseline data, whereas ISFUC constitutes the subsequent follow-up initiated on 4 April 2025. Moreover, participant recruitment and data collection have not been completed and are expected to be finished by 11 June 2026. Preliminary findings on mortality and health outcomes related to non-communicable disease risk factors are expected by late July 2026 and will be limited to descriptive statistics and initially available linked data following basic data cleaning procedures. The baseline cohort for the ISFUC comprises participants recruited in the 2021 STEPS survey, the eighth and most recent STEPS round in Iran [27]. STEPS 2021 was a nationally representative cross-sectional baseline survey undertaken in early 2021 among Iranian adults aged 18 years and older across all 31 provinces. The survey employed a systematic cluster random sampling design, through which 28,821 participants were selected from 3,176 clusters in both rural and urban areas nationwide. In accordance with WHO methodology and national priorities, STEPS 2021 consisted of three sequential steps. The first step involved interviewer-administered questionnaires focusing on sociodemographic characteristics and major metabolic, nutritional, and behavioral risk factors, injury history, health-related quality of life, as well as selected NCD-related medical history. A total of 27,874 participants completed this step. In the second step, 27,745 participants completed standardized physical assessments, encompassing height, weight, waist and hip circumference, and blood pressure. The final step involved 18,119 people aged ≥25, who provided blood and urine samples. The ISFUC study emphasizes on longitudinal assessment over a 5-year follow-up period concerning mortality, healthcare utilization, injuries, reproductive health, fertility-related outcomes, and population growth attitudes.

### Follow-up procedures and participant selection

This follow-up study was initiated following ethical approval five years after the establishment of the baseline cohort, with follow-up commencing in April 2025. All participants who completed the first step of the 2021 STEPS survey (N = 27,874) were eligible for inclusion in the present study. From the 27,874 eligible participants who completed Step 1 of the 2021 STEPS survey, 14,010 individuals will be selected using a disproportionate stratified random sampling approach, stratified by province.To preserve national representativeness and ensure adequate provincial-level representation, a minimum sampling threshold will be determined for each province based on the smallest provincial sample proportion in the STEPS 2021 survey. Consequently, smaller provinces will be sampled at relatively higher fractions, whereas more populous provinces will be sampled at lower fractions. All analyses and results in following reports will be weighted based on the 2016 national census, taking into account province, age, sex, and place of residence, in order to ensure the generalizability of the findings to the Iranian population. Follow-up will be conducted through structured telephone interviews by trained staff using provided smartphones with voice recording ability, subsequent to obtaining renewed verbal consent and strictly adhering to confidentiality protocols. Participants will be contacted between 9:00 a.m. and 8:00 p.m. Upon each call, interviewers will introduce themselves, elucidate the study objectives, and inform participants that the interview will last approximately 5–10 minutes. Participants will then be asked to confirm their identities and confirm their availability for participation.

**Fig 1:**
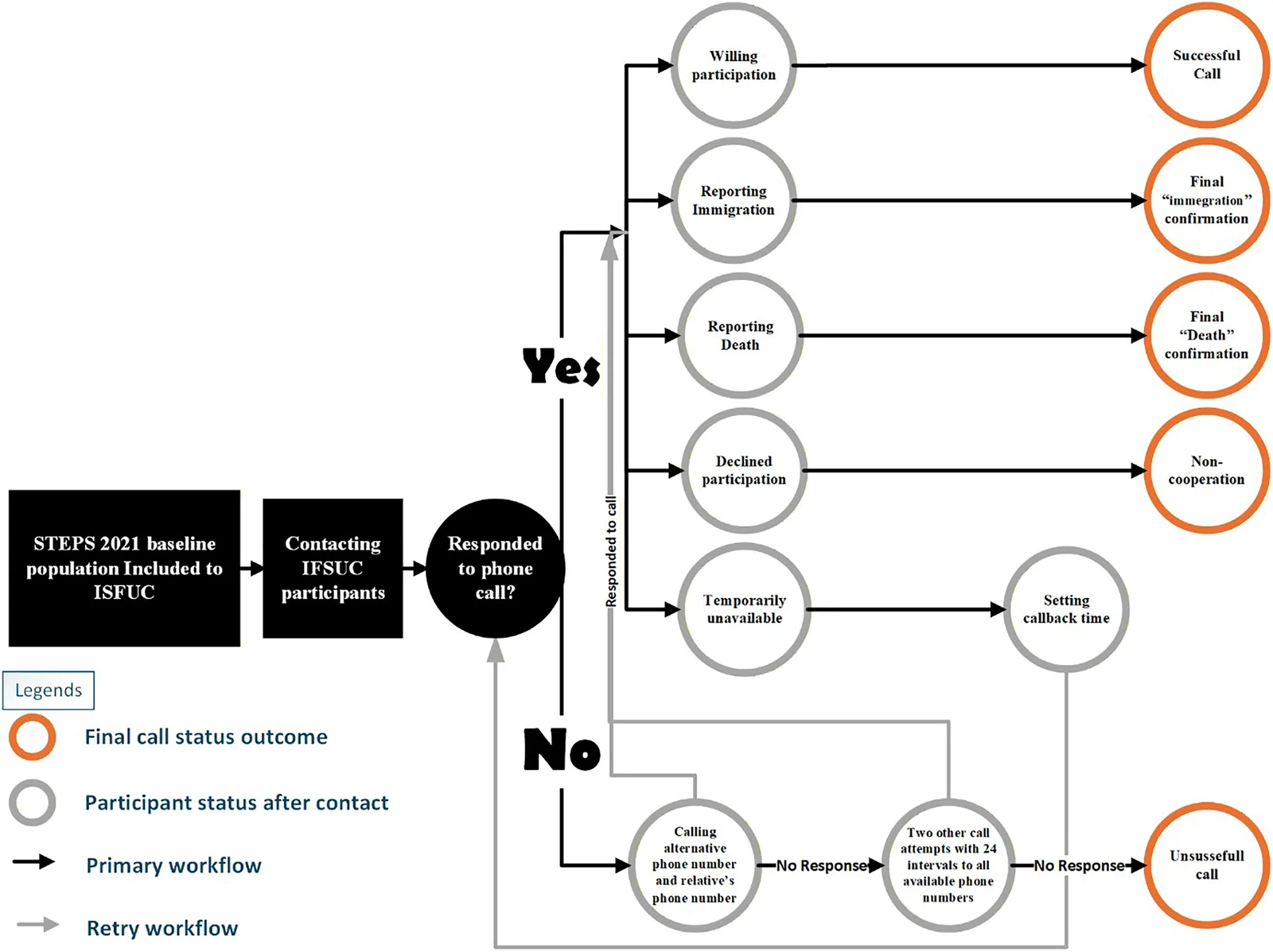
Study flowchart. Abbreviations: STEPS: STEPwise approach to NCD risk factor surveillance; ISFUC: IRAN-STEPS Follow-Up Cohort.

As illustrated in Fig 1, call outcomes are categorized into six predefined groups according to participant responsiveness. A successful call is defined as contact with an eligible participant who consents to participate and completed all sections of the questionnaire. Participants who migrated outside Iran or are confirmed to be deceased by relatives will be documented accordingly, with completion limited to the call-status form. Non-cooperation denotes those who respond the call but declines participation, for whom only call-status information is recorded. An unsuccessful call is defined as a wrong number or a failure to establish contact after three attempts, each made at a different time of day and spaced at least 24 hours apart. In such cases, alternative contact numbers and, subsequently, relatives’ contact numbers will each be attempted. Finally, when participants are temporarily unavailable, a preferred callback time will be arranged and recorded in the call-status form to enable future follow-up. Standardized call-status categories are utilized for each contact number to guarantee uniform reporting and rigorous oversight of response results.

### Variables and questionnaires

Prior to the initiation of the study and interviewer recruitment, an expert panel discussion was conducted to determine the study questions and their details. At this stage, following a review of the scientific literature and preliminary interviews with health experts and specialists, key indicators were identified and developed to assess major health outcomes, including hospitalization, physician visits, and mortality. Based on these indicators, the initial set of questions and a data collection checklist were developed. After drafting the questions, the questionnaire was evaluated to ensure that the wording and terminology were appropriate and comprehensible for the general population. In addition, an interviewer guide was prepared alongside the data collection checklist to provide necessary clarifications for questions requiring further explanation. All variables and corresponding questions are presented in S1 Table in detail.

#### Demographics.

For demographic variables, participants will be asked about residential mobility during the preceding five years, defined as intercity migration or transitions between urban and rural areas; relocations within the same city or village were not considered. Current marital status is classified as never married, married, divorced, separated without formal divorce, widowed, engaged, or unwilling to respond. Divorce is defined as legal separation, whereas separation without divorce required documentation of the duration of separate living. Employment status refers to the participant’s primary occupation during the past five years. Job classification is based on the main occupation and the institution responsible for insurance coverage.

#### Death.

Information collected from respondents will include their relationship to the deceased participant, the month and year of death, and the reported cause of death. When the exact date is unknown, an approximate date will be recorded.

Mortality data, including date of death, cause, and underlying cause will additionally be obtained through linkage of the STEPS dataset with the Iranian Ministry of Health death registry, conducted based on official administrative correspondence with the Network Management Center of the Ministry of Health and Medical Education (deathregistry.behdasht.gov.ir) using national identification numbers, and causes of death will be confirmed by forensic specialists in accordance with legal procedures.

#### Self-reported diseases.

Participants will be asked whether a physician or healthcare worker has diagnosed hypertension, diabetes, or gestational diabetes (for married female participants). For affirmative responses, information on current medication use for hypertension or diabetes during the preceding 12 months will be recorded

#### Outpatient care.

Outpatient care utilization during the past 12 months will be assessed, including diagnoses, treatments, and injury-related services. Participants will be asked whether they have visited a physician’s office or clinic or received home visits; if so, the number of visits for each setting will be recorded. Respondents will identify the most frequently consulted physicians or specialists and the predominant healthcare sector used (public, private, or mixed). Reasons for visits will be documented using a multiple-choice list covering metabolic, cardiovascular, renal, respiratory, musculoskeletal, neurological and psychiatric, oncological, infectious, endocrine, dermatological, gastrointestinal, gynecological, and preventive care conditions, with multiple responses permitted.

For participants with previously reported chronic conditions (e.g., diabetes, hypertension, cardiovascular disease, liver disease, kidney disease, neuropsychiatric disorders), condition-specific follow-up questions will assess the main reasons for related visits (summarized in S1 Table). These items will be administered conditionally, allowing multiple responses, and including a ‘no response’ option. Participants reporting cancer screening will be asked to specify the type of screening, reflecting national programs for breast, cervical, and colorectal cancer among females and colorectal and prostate cancer among males.

#### Inpatient care and emergency department admissions.

Participants will report whether they experienced any hospital or emergency admissions over the preceding five years and, if so, the total number of admissions, the type of facility (public or private sector hospital), and the duration (in days) will be recorded. Long-term hospitalization is defined as admission to a hospital or emergency department for more than 24 hours for medical care, diagnostic procedures, minor surgeries, or intensive care.

#### Injuries and accidents.

Road traffic injuries will be assessed by recording any vehicle-related accidents in the past year, including the total number of accidents. For each accident, respondents will report whether healthcare services were required, the number of related outpatient visits, and whether hospitalization exceeding 24 hours occurred. Participants also report their role in each accident—driver, passenger, or pedestrian—and frequencies will be recorded for each role across events. Drivers will further be asked whether they were at fault and, if so, the number of fault-related accidents will be recorded.

Non-traffic-related injuries, including unintentional burns, falls, drowning, electric shock, unintentional poisoning, violence, snake/scorpion/spider bites, animal attacks, blunt trauma, or other causes during the past 12 months, will also be captured. Minor superficial burns are excluded to focus on clinically significant events. Violence is defined as perceived physical, non-physical, or verbal force imposed on an individual.

#### Reproductive health and fertility.

In this section, females of reproductive age (18–50 years) will be asked about total number of children, age at first birth, pregnancy history during the past five years with detailed counts of live births, miscarriages (pregnancy loss <20 weeks), and stillbirths (≥20 weeks), reasons for miscarriage, mode of delivery for children under five years, history of multiple gestations, use of assisted reproductive technologies (ART), menopausal status and age, infertility history (self or spouse), and contraceptive use (including hormonal, barrier, surgical, natural withdrawal, and abstinence methods). Gynecological or midwifery visits with reasons including pregnancy-related problems (e.g., miscarriage, ectopic pregnancy, bleeding), hormonal disorders (e.g., menopausal symptoms, polycystic ovary syndrome), fertility problems, sexually transmitted and genital infections including human immunodeficiency virus (HIV), gonorrhea, chlamydia, and HPV, urinary or vaginal fungal/bacterial infections, postpartum complications (e.g., postpartum depression, breastfeeding problems), pelvic pain or uterine structural abnormalities, endometriosis, gynecological surgeries, miscarriage-related issues, complications of contraceptive methods, routine checkups, or other reasons.

Married male participants will be asked regarding their total number of children, number of live births during the past five years, history of using ART, current fertility problems affecting the participant or spouse, and current contraceptive use will be recorded, including male methods (e.g., condom, vasectomy, withdrawal, fertility awareness) and spouse-related methods (e.g., oral contraceptives, injectable or implantable hormones, intrauterine device (IUD), tubal ligation, abstinence), with vasectomy and tubal ligation defined explicitly. Moreover, participants will report any specialist visits during the past five years for male health conditions, allowing multiple reasons, including infertility, hormonal disorders (e.g., testosterone abnormalities), sexually transmitted infections (e.g., HIV, gonorrhea, chlamydia), urinary disorders, sexual dysfunction, prostate disease, urinary stone disease, and routine check-ups.

#### Childbearing and population growth attitudes.

The youthful population and protection of the family law, enacted in October 2021 as a government initiative to encourage childbearing, comprises 73 articles and 81 notes and primarily includes incentives to support marriage and childbearing, as well as restrictions on access to family planning and reproductive health services [32]. Married male and female participants’ awareness of this law was assessed on a graded Likert-type scale from ‘not at all’ to ‘very much.’ Their knowledge of the law’s benefits was then evaluated. Within the ISFUC study, we sought to evaluate participants’ attitudes toward this policy. Participants who report being aware of these initiatives will be further asked whether they have utilized any related benefits, to specify the type of benefit if applicable, and to report their level of satisfaction with the benefits received. Moreover, participants will indicate the ideal number of children for a family and will identify factors that could encourage higher fertility, allowing multiple responses, including financial incentives, reduced infertility costs, housing support, free educational services, extended paternal leave, childcare support, job security, affordable kindergartens, reduced pregnancy and delivery costs, paternal involvement in childcare, marriage and fertility incentives, salary continuation during maternity leave, insurance for non-employed mothers, other governmental supports, or none. Fertility intentions will be assessed separately for female and male, capturing current pregnancy status and planned timing of childbirth. Participants reporting no intention to have children will be subsequently asked to report reasons for not pursuing childbearing in the past five years, including economic, occupational, social pressure, familial, medical, age-related, migration-related, or other factors; multiple selections were permitted.

### Face and content validity

Face validity was assessed qualitatively by a panel of approximately 20 participants, including methodologists, subject-matter experts, and members of the target population. They reviewed the checklist for clarity, simplicity, and comprehensibility of wording and suggested modifications where necessary [33,34]. Content validity was assessed by a panel of 10 experts, comprising methodologists, subject-matter experts, and representatives of the target population, using the Content Validity Ratio (CVR) and the Content Validity Index (CVI). For CVR, experts rated each item on a three-point Likert scale (“essential”, “useful but not essential”, “not essential”) and the CVR was calculated using Lawshe’s method [35]; items with CVR < 0.62 (based on 10 experts) were revised and re-evaluated, and removed if still below the acceptable threshold. CVI was calculated using the Waltz and Bausell approach by rating each item for relevance, clarity, and simplicity on a four-point Likert scale. Item-level CVI (I-CVI) was computed as the proportion of experts assigning a score of 3 or 4; items with I-CVI ≥ 0.79 were retained, 0.70–0.79 were revised, and < 0.70 were removed [33]. Scale-level CVI (S-CVI/Ave) was calculated as the average of I-CVI values across all items, and an S-CVI/Ave ≥ 0.80 was considered acceptable [33].

### Data management, quality assurance

Standardized data management and quality control protocols will be implemented to ensure accuracy and completeness. Data will be entered directly into electronic tablets during interviews and securely uploaded to a central server within one week. To minimize the risk of data loss due to hardware malfunctions, communication failures, or unintentional deletion, regular data backups will be conducted systematically, and backup storage devices will be maintained at both field and central levels. In addition, each interviewer will be provided with both a laptop and a mobile device to ensure continuity of data transfer and storage in case of technical problems with one device. Quality control will encompass random audits of audio-recorded interviews compared to entered data, weekly assessments of interviewer performance accompanied by feedback and retraining, random field supervision, and periodic interim analyses to identify and resolve issues. In addition, weekly back-check calls will be conducted for a random sample of completed interviews to assess participant satisfaction and to re-administer selected questionnaire items; responses will be cross-verified against recorded forms, and in cases of discrepancies, the interviewer’s forms will be subjected to more intensive review during the same week. Interviewers will submit weekly activity reports, while the executive manager will engage in daily communication with the field team.

### Outcomes

In this cohort study, outcomes were defined a priori based on the structured questionnaire domains and were aligned with the main objectives of assessing health status, healthcare utilization, and fertility-related behaviors and attitudes over time. The primary outcomes included mortality, defined as all-cause death occurring within five years after the baseline assessment; incident hypertension and incident diabetes mellitus were defined as new cases of physician-diagnosed hypertension or diabetes or initiation of antihypertensive or antihyperglycemic drugs reported during follow-up among participants free of the respective condition at baseline. Incidence estimates will therefore be based on self-reported current disease status and medication use at follow-up among participants without the corresponding condition at baseline. Healthcare utilization as a primary outcome was defined as the need for inpatient care within five years after baseline, including any physician or clinic visits, home physician visits, emergency department admissions, or hospitalizations, as well as the frequency of these encounters during the follow-up period.

The secondary outcomes encompassed a broad range of health-related, reproductive, and behavioral indicators assessed longitudinally. These included patterns of outpatient care (type of provider, public versus private sector use, and reasons for visits), injury-related outcomes such as traffic accidents and other unintentional or intentional injuries requiring medical attention, and detailed hospitalization characteristics including causes, type of facility, and duration of long-term admissions. Additional secondary outcomes included reproductive health indicators and fertility-related behaviors and attitudes, with sex-specific measures for women and men, such as pregnancy history, infertility, contraceptive use, utilization of reproductive health services, fertility intentions, and perceptions and use of governmental childbearing support policies.

### Statistical analysis plan (SAP)

Data cleaning and validation will be conducted by two independent analysts. Any discrepancies will be resolved through review by a third analyst, and the finalized dataset will be stored securely with restricted access to preserve confidentiality and data integrity.

Descriptive analyses will be used to summarize baseline characteristics and follow-up outcomes. Continuous variables will be expressed as weighted mean (95% CI) or weighted median [95% CI], depending on distributional properties. Categorical variables will be reported weighted percentage (95% CI). The primary time-to-event outcome will be all-cause mortality. Mortality will be analyzed using Cox proportional hazards regression, with time calculated from the baseline STEPS assessment to death. The proportional hazards (PH) assumption for the Cox proportional hazards models will be assessed using Schoenfeld residuals. For common binary outcomes, log-binomial regression or Poisson regression with robust variance estimation will be used to estimate risk ratios (RRs). Logistic regression will be used only if these models fail to converge. Count outcomes, such as number of outpatient visits, hospital admissions, or injury events, will be analyzed using Poisson regression or negative binomial regression in the presence of overdispersion. Ordinal outcomes will be analyzed using ordinal logistic regression, while nominal categorical outcomes with more than two categories will be analyzed using multinomial logistic regression. All regression models will be adjusted for prespecified covariates selected a priori on the basis of the study objectives and available baseline data. These will include age, sex, marital status, province, urban/rural residence, education, occupation, and relevant baseline risk factors, as appropriate for each outcome. Additional covariates may be included based on biological plausibility and prior evidence. Model assumptions will be assessed before final inference. Where necessary, transformations or alternative model specifications will be used.

For missing covariate data, the handling strategy will depend on the proportion and presumed mechanism of missingness. When covariate missingness is between 5% and 10% and considered to be missing at random, complete-case analysis will be performed. However, when missingness exceeds 10%, sensitivity analyses using multiple imputation methods will be conducted. Results obtained from imputed datasets will be compared with complete-case analyses to evaluate the robustness of findings under different missing-data assumptions [36,37].

The final analytical weights for ISFUC will be derived by re-adjusting the original STEPS 2021 sampling weights to reflect the differential sub-sampling probabilities across strata. Consequently, analytical weights will be derived via post-stratification to the 2016 national census. For each stratum will be formed by the cross-classification of province, sex, urban/rural residence, and age category, the weight will be computed as W = N/n, where N is the census population count and n is the number of respondents in that stratum. These weights will adjust for the disproportionate stratified sampling used to select the follow-up cohort and for differential non-response, ensuring that the weighted estimates are representative of the Iranian adult population.

## Discussion

The ISFUC study constitutes the inaugural nationally representative telephone-based cohort in Iran derived from the most recent round of the STEPS survey conducted post-COVID-19 pandemic. By leveraging the 2021 STEPS infrastructure, the cohort enables longitudinal assessment of mortality, incident non-communicable diseases, healthcare utilization, reproductive health, and fertility-related outcomes using standardized baseline clinical, anthropometric, and laboratory data. In light of the escalating burden of NCDs in Iran and the broader Middle East and North Africa (MENA) region [9,10,38], specifically after COVID-19 pandemic, which has substantially influenced NCDs and their associated risk profiles [26], the establishment of a post–COVID-19 nationally representative cohort could yield critical insights for health policy and public health planning.

Several large previous undertaken cohort studies in Iran, including the PERSIAN cohort [23] and the ICS [24], have substantially contributed to epidemiological research in the country. However, many earlier cohorts were either not nationally representative, lacked comprehensive baseline assessments of NCD-related factors, or were conducted before the COVID-19 pandemic. In contrast, ISFUC is based on the 2021 STEPS survey, which introduced several methodological and clinical enhancements compared with earlier STEPS rounds, including the assessment of eGFR, urine albumin-to-creatinine ratio, and cancer screening indicators had been added [26,27], that can help policymakers to implant health policies. Furthermore, the post–COVID-19 timing of the cohort is particularly relevant, as the pandemic has been associated with disruptions in NCD prevention and healthcare services, changes in risk factor profiles, and potential long-term effects on NCD outcomes [26,39,40]. Specifically, accumulating evidence suggests that post–COVID-19 conditions (“Long COVID”) may increase the risk of incident diabetes, hypertension, chronic kidney disease, and cardiovascular diseases [41].

To keep the present study’s bias at minimum, several methodological considerations were addressed in the study design. As the baseline STEPS study was nationally representative, to minimize selection bias and preserve national representativeness, a random sub-sample of the participants from the original STEPS cohort was included by random selection across all 31 provinces. Specifically, although approximately 50% of eligible STEPS participants were selected for follow-up using a disproportionate stratified random sampling approach (stratified by province), national representativeness is preserved through post-stratification weighting and less populous provinces sampled at higher fractions and more populous provinces at lower. All results will be weighted based on the 2016 national census, incorporating province, age, sex, and place of residence, to ensure that findings remain generalizable to the Iranian adult population. Moreover, Standardized questionnaires, interviewer training, structured interview protocols, and multi-level quality control procedures are also implemented to reduce information bias and improve data consistency [42]. In addition, systematic follow-up procedures are adopted to improve participant retention and follow-up completeness and reduce loss to follow-up.

### Strengths and limitations

A major strength of ISFUC is the integration of multiple outcome domains that are directly relevant to current public health priorities in Iran. With a large national sample spanning all 31 provinces, it supports the generalizability of the findings to the Iranian population. The ISFUC enables comprehensive linkage of demographic, metabolic, nutritional, behavioral, anthropometric, and biomarker data with mortality and major NCD outcomes, thereby expanding current evidence in the Iranian population. In addition to conventional epidemiological outcomes such as mortality, incident hypertension, and diabetes, the cohort systematically captures outpatient and inpatient healthcare utilization, providing policy-relevant indicators of healthcare demand, access, utilization patterns, and system burden. Therefore, beyond its role as an epidemiological cohort, ISFUC also serves as a valuable health services research platform by enabling assessment of patient interaction with healthcare systems and patterns of healthcare utilization. These measures offer important insights for health system planning, resource allocation, and evaluation of service delivery, particularly in the context of rising NCD prevalence and constrained healthcare resources. In addition, the cohort also enables assessment of traffic- and non-traffic-related injuries at the population level. Furthermore, Iran is currently experiencing an epidemiological and demographic transition characterized by a high burden of non-communicable diseases alongside declining fertility rates and population aging. This “double burden” may have substantial long-term implications for population health, healthcare demand, and socioeconomic development. Thus, as the first nationally representative cohort of its kind, ISFUC provides a unique opportunity not only to evaluate fertility patterns and the population-level impact of recent population growth policies, but also to investigate the potential relationships between NCDs, related risk factors, and fertility outcomes within the STEPS framework. Moreover, the study relied primarily on telephone interviews which provide a cost-efficient approach for follow-up data collection in low-resource settings [29,30]. However, experience with telephone-based cohort studies in low-income countries remains limited worldwide [30].

Notwithstanding these strengths, several limitations inherent to the study design should be acknowledged. First, the follow-up duration may be insufficient to capture the full incidence of diseases with long latency periods such as diabetes complications. Furthermore, these data rely in part on self-reported information, specifically variables such as inpatient care over a 5-year period, may introduce recall bias or misclassification. However, these limitations were partially mitigated through standardized instruments and interviewer training. In addition, the assessment of hypertension and diabetes based on self-report, rather than objective measurements as in the baseline STEPS protocol, may lead to underestimation of their true prevalence. Therefore, findings should be interpreted with caution, particularly when comparing follow-up estimates with baseline STEPS data. Moreover, although loss to follow-up remains a concern, systematic re-contact protocols were implemented to enhance follow-up comprehensiveness. Furthermore, loss to follow-up may exhibit a social pattern, potentially linked to participants’ limited access to communication resources and underlying socioeconomic constraints. Given the telephone-based design of the present cohort, differential follow-up may also arise from the “digital divide,” potentially leading to underrepresentation of marginalized populations with unstable or limited access to telecommunications services [43]. To mitigate this source of bias, multiple contact attempts at different times of day and the use of alternative contact numbers, when available, were implemented to improve participant accessibility and follow-up completeness. In addition, culturally shaped reporting behaviors and social desirability effects may introduce response bias. Consequently, future analyses should interpret findings with caution and consider conducting sensitivity and subgroup analyses to assess the potential impact of differential follow-up, cultural factors, and digital divide–related bias. Taken together, the ISFUC cohort offers a valuable, policy-relevant platform for monitoring emerging public health challenges in Iran, while its methodological limitations underscore the need for interpreting results with caution.

## Conclusion

ISFUC is the first nationally representative Iranian cohort to link comprehensive baseline data from the 2021 STEPS survey, the most recent national STEPS round, with structured telephone-based longitudinal follow-up across all 31 provinces. The 2021 baseline survey and subsequent follow-up were conducted following the onset of the COVID-19 pandemic, providing an opportunity to examine health outcomes within a changing epidemiological context. ISFUC provides a robust and scalable framework for longitudinal assessment of health outcomes, healthcare utilization, and fertility-related behaviors in Iran. In particular, the integration of conventional NCD outcomes with reproductive health, childbearing behaviors, and healthcare utilization indicators addresses an underexamined public health need in Iran. This integrated scope, together with the COVID-19-related context of the baseline survey, represents the principal contribution of ISFUC and distinguishes it from earlier Iranian cohort platforms. This protocol study outlines the design, objectives, methodology, generalizability, and strengths and limitations of the ISFUC cohort, enabling researchers worldwide to critically appraise and engage with the study framework prior to completion of follow-up.

## Supporting information

S1 Table Study questions and sub-item questions across domains. (DOCX)

## Abbreviations

NCDs: Non-communicable diseases
BMI: body mass index
STEPS: The STEPwise Approach to NCD Risk Factor Surveillance
WHO: The World Health Organization
IraPEN: The Iran Package of Essential Noncommunicable Diseases
WHO-PEN: The WHO Package of Essential Non-communicable Diseases
CKD: chronic kidney disease
TCGS: The Tehran Cardiometabolic Genetic Study
PERSIAN: The Prospective Epidemiological Research Studies in IrAN
ICS: The Iran Cohort Study
eGFR: estimated glomerular filtration rate
ISFUC: The IRAN-STEPS Follow-Up Cohort
NIHR: The National Institute of Health Research
HPV: human papillomavirus
HDL-C: high-density lipoprotein cholesterol
ALT: alanine transaminase
BUN: blood urea nitrogen
HbA1c: hemoglobin A1C
PPS: Probability Proportional to Size
ART: assisted reproductive technologies
HIV: human immunodeficiency virus
IUD: intrauterine device
CVR: Content Validity Ratio
CVI: Content Validity Index
MENA: The Middle East and North Africa
