# Supplementary material for: Mortality and health outcomes associated with non-communicable disease risk factors: Protocol for the IRAN-STEPS Follow-Up Cohort (ISFUC) based on the 2021 national survey
Source: PLoS One. 2026 Aug 31;21(8):e0356802. doi: 10.1371/journal.pone.0356802 (PMC13529014; doi:10.1371/journal.pone.0356802)
Supplement: Supplementary file 1 [file pone.0356802.s001.docx]

**S1 Table. Study questions and sub-item questions across domains.**

| Variable Domain | Sub-category | Main item questions | Sub-item questions |
| --- | --- | --- | --- |
| Demographics | Residential mobility | No change/Another city/Urban to rural / Rural to Urban / Not willing to answer | - |
|  | Current marital status | Never married / Married / Divorced or separated (living apart without divorce) / Widowed / Engaged / Not willing to answer | Marriage duration (if married) Years (integer; if <1 year recorded as 1) |
|  |  |  | Duration since divorce (if divorced) Years (integer) |
|  |  |  | Duration living apart without divorce (if separated) Years (integer) |
|  | Job status (within past 5 years) | Public-sector employee / Public-sector worker / Private-sector employee / Private-sector worker / Self-employed / Unpaid work / Student / Soldier / Homemaker / Retired / Unemployed due to disability / Unemployed seeking work / Unemployed not seeking / Not willing to answer | - |
| Death | - | Is the participant dead? | Yes / No / Not willing to answer |
|  |  |  | Date of death (year/month) |
|  |  |  | Relationship of respondent to deceased |
|  |  |  | Cause of death (optional) |
| Self-reported Disease | Hypertension | Ever told has hypertension? | Yes / No / Not willing to answer |
|  |  |  | Currently using antihypertensive medication (last 12 months) Yes / No / Not willing to answer |
|  | Diabetes | Ever told has diabetes / high blood glucose? | Yes / No / Not willing to answer |
|  |  |  | Currently using glucose-lowering medication (last 12 months)? Yes / No / Not willing to answer |
|  |  |  | Ever had gestational diabetes? Yes / No / Not willing to answer |
| Outpatient care | Outpatient/home physician visit | Any outpatient visits or home physician visit in past 12 months? | Yes / No |
|  |  |  | Number of home physician visits (past 12 months)? Count (integer) |
|  |  |  | Number of office/clinic visits (past 12 months)? Count (integer) |
|  |  |  | Five most visited physician/specialist |
|  | Sector of most outpatient care | Only public / Only private / More public–less private / Less public–more private / Public and private equally / Not willing to answer | - |
|  | Outpatient care Reason(s) for visit | Diabetes / Hypertension / Heart disease / Stroke sequelae / Osteoarthritis / Rheumatoid arthritis / Liver problems / Mental health–neurology–sleep problems / Cancer screening / Cancer care or treatment / Asthma / Other chronic respiratory disease (non-asthma) / Kidney disease–problems / Back pain / Fracture due to traffic accident / Fracture (non-traffic) / Osteoporosis / Obesity / Women’s health problems / Men’s health problems / Dermatological problems / ENT / Gastrointestinal / Eye problems / High blood lipids / Periodic checkup–follow-up / Infectious disease / Hypothyroidism or hyperthyroidism / Other (specify) / Not willing to answer. | Diabetes visits reason(s)? Including Kidney problems / Dialysis / Eye problems / Numbness/tingling hands/feet / Diabetic foot ulcer / Diabetic foot care–complication / Heart problems / Checkup–follow-up / Other diabetes complications / Other (specify) / Not willing to answer |
|  |  |  | Hypertension visit reason(s)? Including Hypertension complications (e.g., stroke, renal failure, heart disease, eye damage) / Checkup–follow-up / Other (specify) / Not willing to answer |
|  |  |  | Heart disease visits reason(s)? Including Myocardial infarction / Heart failure / valvular disease / Arrhythmia / pectoris angina / Cardiac assessments (ECG/echo/stress test/…) / Hypertension / Cardiopulmonary edema / Other (specify) / Not willing to answer |
|  |  |  | Liver visit reason(s)? Including Fatty liver / Cirrhosis / Hepatitis / Elevated liver enzymes / Checkup–follow-up / Other (specify) / Not willing to answer |
|  |  |  | Kidney visits reason(s)? Including Kidney stones / Kidney & urinary tract infection / Proteinuria or nephrotic syndrome / Elevated blood creatinine / Dialysis / Checkup–follow-up / Acute kidney failure / Chronic kidney failure / Other (specify) / Not willing to answer |
|  |  |  | Mental health/neurology/sleep visit reason(s)? Including Depression / OCD / Anxiety / Parkinson’s / Alzheimer’s / Multiple sclerosis / Headache / Sleep disorders / Checkup–follow-up / Other (specify) / Not willing to answer |
|  |  |  | Type of cancer screened for (if cancer screening selected)? Free text (e.g., breast/cervix/colon/prostate/…) |
| Injuries & accidents | Traffic accident  (past 12 months) | Any traffic accident in past 12 months? | Yes / No / Not willing to answer |
|  |  |  | Number of traffic accidents (past 12 months)? Count (integer) |
|  |  |  | Role in accident? driver (count) Count (integer); At-fault in traffic accident? Yes / No / Not willing to answer |
|  |  |  | Role in accident? passenger (count) Count (integer) |
|  |  |  | Role in accident? pedestrian (count) Count (integer) |
|  |  | Any accident needing clinic/hospital visit/transfer | No visit needed / Yes / Not willing to answer |
|  |  |  | Number of accidents needing clinic/hospital visit/transfer Count (integer) |
|  |  | Any accident resulting in hospitalization >24h | Yes / No / Not willing to answer |
|  |  |  | Number of accidents resulting in hospitalization >24h Count (integer) |
|  | Non-traffic injury/accident | Any non-traffic injury/accident in past 12 months | Yes / No / Not willing to answer |
|  |  |  | Type(s) of non-traffic injury/accident? Including Unintentional burn / Fall & ground-level fall / Drowning / Electrocution / Unintentional poisoning / Violence / Snake–scorpion–tarantula bite/sting / Animal attack / Trauma/ Other (specify) / Not willing to answer |
| Inpatient Care and Emergency Department Admissions |  | Any hospitalization/ED admission in past 5 years | Yes / No / Not willing to answer |
|  |  |  | Number of hospitalizations/ED admissions (past 5 years)? Count (integer) |
|  |  |  | Hospital type? Private hospital (count) / Public hospital (count) |
|  |  |  | Top Five Main causes |
| Reproductive Health and Fertility | Females | Total number of children | Count (integer) |
|  |  | Age at first birth | Years (integer) |
|  |  | Any pregnancy in past 5 years | Yes / No / Not willing to answer |
|  |  |  | Number of pregnancies (past 5 years)? Count (integer) |
|  |  |  | Number of live births (past 5 years)? Count (integer) |
|  |  |  | Number of miscarriages (past 5 years)? Count (integer) |
|  |  |  | Number of stillbirths (past 5 years)? Count (integer) |
|  |  |  | Miscarriage reasons (counts; if miscarriage>0)? Spontaneous (count) / Congenital anomaly (count) / Accident-related (count) / Other (count) / Not willing to answer |
|  |  |  | Delivery type for children under 5 years? Vaginal (count) / Cesarean (count) / Not willing to answer |
|  |  |  | Any multiple gestation (twins or more)? Yes / No / Not willing to answer |
|  |  |  | Number of multiple gestation events? Count (integer) |
|  |  | Using any ART methods? | Yes / No / Not willing to answer |
|  |  | Menopause / hysterectomy status | Not menopausal / Menopausal / Had hysterectomy / Not willing to answer |
|  |  |  | Age at menopause (if menopausal)? Years (integer) |
|  |  | Current infertility (self or spouse) | Yes / No / Not willing to answer |
|  |  |  | Infertility cause (self) self-report |
|  |  |  | Infertility cause (spouse) self-report |
|  |  | Currently using contraception | Yes / No/ Not willing to answer |
|  |  |  | Contraception method(s) (multi-select)? Oral pills / Patch / Injection / Implant / Calendar method / Body temperature method / Condom / Diaphragm / Cervical cap / IUD / Tubal ligation / Withdrawal / Partner vasectomy / No sexual intercourse / Not willing to answer |
|  |  | Any visit to OB-GYN/midwife in past 5 years | Yes / No / Not willing to answer |
|  |  |  | Reason(s) for OB-GYN/midwife visit? Menstrual problems / Pregnancy problems (miscarriage/ectopic/bleeding) / Hormonal problems (menopause symptoms/PCOS) / Infertility/ STIs & genital infections / Urinary & vaginal fungal/bacterial infections / Postpartum problems / Pelvic pain/structural uterine problems / Endometriosis / Gynecologic surgery / Miscarriage problems / Contraception complications / Routine checkup / Other (specify) / Not willing to answer |
|  | Males | Total number of children | Count (integer) |
|  |  | Total number of children under 5 years | Count (integer) |
|  |  | ART use | Yes / No / Not willing to answer |
|  |  | Current infertility (self or spouse) | Yes / No / Not willing to answer |
|  |  |  | Infertility cause (self) Free text |
|  |  |  | Infertility cause (spouse) Free text |
|  |  | Currently using contraception (self or partner) | Yes / No / Not willing to answer |
|  |  |  | Contraception method(s) (multi-select) Calendar method / Body temperature method / Condom / Vasectomy / Withdrawal / Partner oral pills / Partner patch / Partner injection / Partner implant / Partner diaphragm / Partner cervical cap / Partner IUD / Partner tubal ligation / No sexual intercourse / Not willing to answer |
|  |  | Any specialist visits for male-health issues in past 5 years | Yes / No / Not willing to answer |
|  |  |  | Reason(s) for male-health specialist visit (multi-select)? Infertility problems / Hormonal (testosterone) disorders / STIs / Urinary disorders (UTI, incontinence) / Erectile dysfunction/sexual problems / Prostate problems / Urinary stone disease / Routine checkup / Other (specify) / Not willing to answer |
| Childbearing and Population Growth Attitudes | Youth population | Awareness of government childbearing initiatives? | Not at all / Low / Somewhat / High / Very high / No opinion / Not willing to answer |
|  |  | Aware of benefits of the “Youth Population” law? | Yes / No / Not willing to answer |
|  |  | Used benefits of the “Youth Population” law? | Yes / No / Not willing to answer |
|  |  |  | Type of benefit used (if used)? Specify |
|  |  |  | Satisfaction with used benefits (if used)? Yes / No / Not willing to answer |
|  |  | Ideal number of children for a family | 1 / 2 / 3 / 4 or more / Not willing to answer |
|  |  | Factors encouraging higher fertility (multi-select)? | Direct financial payments / Reduced infertility costs / Housing support / Free education services / Extended paternal leave / Childcare support / Job security / Lower-cost kindergartens / Reduced pregnancy care costs / Reduced delivery care costs / Father involvement in childcare / Marriage incentives / Childbearing incentives / Salary during maternity leave / Insurance for non-employed mothers / Other (specify) / None / Not willing to answer |
|  |  | Fertility intention? | Currently Expecting a child / Yes, within next year / Yes, within <5 years / Yes, in >5 years / No / Not willing to answer |
|  |  |  | Reasons for not deciding to have children? (multi-select; married, childless; if not intended to have a children)? Economic conditions / Job conditions / Social pressure / Family pressure / Concerns about children’s future / Medical issues or infertility / My illness / Spouse’s illness / Spouse’s menopause / Older age / Intention to immigrate / Other (specify) / Not willing to answer |

Abbreviations: OB-GYN: Obstetrics and Gynecology; ENT: Ear, Nose, and Throat; ECG: Electrocardiogram; OCD: Obsessive-Compulsive Disorder; ED: Emergency Department; ART: Assisted Reproductive Technology; IUD: Intrauterine Device; PCOS: Polycystic Ovary Syndrome; STIs: Sexually Transmitted Infections.
